# Parenting Amid Shadows: Exploring the Child-Rearing Experiences of Wives of Individuals With Alcohol Use Disorder

**DOI:** 10.7759/cureus.58105

**Published:** 2024-04-12

**Authors:** Akhil P. Joseph, Anithamol Babu, L T Om Prakash

**Affiliations:** 1 Department of Sociology and Social Work, Christ (Deemed to be University), Bengaluru, IND; 2 School of Social Work, Marian College Kuttikkanam Autonomous, Kuttikkanam, IND; 3 School of Social Work, Tata Institute of Social Sciences Guwahati Off-Campus, Jalukbari, IND

**Keywords:** children, wives, family dynamics, coping strategies, parenting, alcohol use disorder

## Abstract

Introduction: The pervasive impact of alcohol use disorder (AUD) within families, particularly on parenting roles in Kerala, India, necessitates an in-depth exploration. This study aims to uncover the unique challenges and coping strategies employed by wives of individuals with AUD against a backdrop of societal stigma and economic hardship.

Methodology: This study, employing a qualitative narrative research design, delves into the experiences of 30 wives of men with AUD in Kerala. The study delves into the complexities of navigating parenting responsibilities amid challenges related to AUD, employing in-depth interviews with the aid of a semi-structured interview guide conducted in Malayalam.​​​​​ The researchers used narrative analysis to extract the themes related to coping mechanisms, resilience, and the impact on children's psychological health.

Results: The study highlights significant emotional and social burdens on wives, including solo parenting, financial strain, and social stigma. Despite these challenges, the resilience and adaptive strategies of these women stand out, with extended family support, community resources, and personal beliefs playing crucial roles in their coping mechanisms. The study points out variations in coping strategies across different socio-economic and educational backgrounds.

Implications: The findings underscore the necessity for comprehensive support systems sensitive to the socio-demographic differences among families affected by AUD. Tailored interventions that enhance access to professional support, foster community solidarity, and provide economic assistance are critical. Moreover, efforts to reduce stigma and promote understanding are essential for improving the psychological health and overall quality of life of these families.

## Introduction

The impact of alcohol use disorder (AUD) within family systems, especially in Kerala, India, introduces a multidimensional dilemma that not only affects the individual struggling with the disorder but also places a significant burden on family members. The pervasive influence of AUD on family dynamics, including but not limited to the disruption of parenting roles, the strain on spousal relationships, and the alteration of child-parent interactions, necessitates a thorough investigation [1,2]. Kerala, characterized by its unique socio-cultural landscape and the highest per capita alcohol consumption in India, provides a critical backdrop for examining the nuanced ways in which wives of individuals with AUD manage parenting [3,4]. This exploration is imperative, given the significant mental health implications for caregivers and the children involved [5,6]. Research has consistently highlighted the adverse effects of parental AUD on children. This includes a heightened risk of emotional issues such as anxiety and depression, behavioral problems like aggression and withdrawal, and academic challenges, leading to poor performance and decreased school attendance [7,8]. However, the specific experiences of wives bearing the dual burden of caregiving for their husbands with AUD and parenting their children remain underexplored, particularly within the South Asian context. These women often find themselves in a precarious position, attempting to shield their children from the chaos engendered by AUD while managing the societal stigma and economic hardships that accompany their husband's condition. The resilience and coping strategies employed by these women in the face of such adversity are crucial areas of inquiry, as they have profound implications for the mental health and well-being of the entire family.

The societal and familial expectations placed on women in Kerala to maintain familial harmony and protect the family's reputation significantly intensify the adversities encountered by these wives [9,10]. Moreover, the cultural norms surrounding alcohol consumption, gender roles, and family secrecy further exacerbate these wives' psychological stress and isolation. This scenario is compounded by the lack of adequate support systems and resources for families affected by AUD, leaving these women to manage their issues [11]. Understanding the specific context of Kerala, with its distinct socio-cultural and economic factors, is crucial in addressing the broader implications of AUD on families. Therefore, this study addresses two main questions: How do these wives perceive the impact of AUD on their parenting responsibilities and the challenges they face? Moreover, what coping strategies and support systems do they employ, considering variations across socio-demographic backgrounds?

## Materials and methods

Research design

This study employed a qualitative narrative research design to explore the intricate experiences of wives of individuals with AUD in Kerala, India, primarily focusing on their parenting roles. Narrative research, situated within the constructivist paradigm, facilitates a comprehensive understanding of individual experiences through the narratives individuals share about their lives, particularly in contexts that are culturally and emotionally complex [12].

Setting and participants

The researchers conducted the study in Kerala, India, known for its distinct socio-cultural environment and significant incidence of AUD [13]. They focused on the wives of individuals with AUD, drawing participants from urban and rural Kerala to capture a broad spectrum of experiences. Through purposive sampling, they chose participants who were willing to share their experiences, had been living with their AUD-affected husbands for the last year, and had at least two children younger than 15.

Data collection

Data collection involved in-depth, semi-structured interviews, specifically chosen to delve into the personal narratives of wives of AUD individuals, balancing between guided focus and the flexibility to uncover unexpected insights. The researchers designed interviews to gather narratives on the impact of their husband's AUD on family life and coping strategies. Interviews were conducted in Malayalam to ensure authenticity, and the interviews were audio-recorded with consent and transcribed verbatim for analysis.

Data analysis

Data analysis was conducted through narrative analysis, focusing on the stories shared by participants to uncover the underlying themes and patterns in their experiences with parenting in the context of a husband's AUD. This approach involved meticulously examining the narratives for content, structure, and context, enabling a deep understanding of how these wives construct their identities and coping strategies within their challenging environments [14].

Ethical considerations

This study adhered to ethical standards, securing approval from the Institutional Review Board (IRB) before commencement. Informed consent was obtained from all participants, ensuring they were fully aware of the study's nature, its potential risks and benefits, and their rights, including the right to withdraw at any point. The study rigorously maintained confidentiality and anonymity, removing all identifying information when presenting the findings.

## Results

Socio-demographic characteristics

The study encompassed a diverse group of 30 wives of individuals with AUD residing in Kerala. The ages of the participants ranged from 28 to 55 years. The educational levels varied significantly among the participants; most had completed secondary education, nearly one-third held undergraduate degrees, and the remaining had attained education up to primary school or had no formal education. However, half of the participants were employed in formal sectors or through informal work, while the other half were homemakers. The participants' marriages ranged from five to 30 years. The number of children per participant ranged from one to four, with the majority having two children. Socio-economic status, assessed based on income, showed that more than one-third of the participants belonged to lower socio-economic backgrounds, half belonged to the middle, and only three belonged to the upper-middle socio-economic strata (Table 1).

**Table 1:** Participants profile

Characteristics		Number
Age	28-40	12
	40-55	18
Education	Secondary education	12
	Undergraduate degrees	9
	Primary education/no formal education	9
Employment status	Employed (formal/informal)	15 (8 informal & 7 formal)
	Homemakers	15
Duration of marriage	5-30 years	30
Number of children	Two	18
	Three or four	12
Socio-economic status	Lower	12
	Middle	15
	Upper-middle	3

Perceived impact on parenting roles and responsibilities

This theme encapsulates the multidimensional challenges faced by wives of individuals with AUD. These challenges range from the immediate logistical and emotional burdens of solo parenting to the broader concerns regarding the family's psychological well-being and financial stability. Participants articulate a profound sense of responsibility and isolation as they manage the complexities of parenting without a supportive co-parenting partner. The emotional and psychological toll on the children is a recurring concern, with mothers deeply worried about the immediate and long-term effects of AUD on their well-being. Financial difficulties further compound these challenges, with the diversion of resources towards alcohol consumption leading to economic strain. The social stigma associated with AUD adds another layer of difficulty, affecting not only the individuals with AUD but also their families, leading to social isolation and additional stress. The emotional and physical welfare of the wives themselves emerges as a significant concern, with many reporting distress and health issues because of their caregiving responsibilities (Table 2).

**Table 2:** Perceived impact on parenting roles and responsibilities

Theme	Sub-theme	Participant Verbatim
Perceived impact on parenting roles and responsibilities	Solo parenting	"I find myself playing the role of both parents. It is exhausting and lonely."
	Emotional and logistical burdens	"Every decision, every responsibility falls on me. From paying bills to attending parent-teacher meetings, I am stretched thin."
	Psychological health of children	"They try to hide it, but I know they are hurting. The confusion and sadness in their eyes break my heart."
	Long-term effects on children	"I am scared of how this will shape them as adults. Will they carry this pain with them?"
	Financial difficulties	"We are constantly juggling bills, and it is a struggle to afford necessities. His addiction is draining our finances."
	Social stigma	"The whispers and stares we get have isolated us. It is not just him suffering; we are all painted with the same brush."
	Emotional and Physical Welfare of Wives	"It is a constant state of worry for me. My health has taken a backseat, but I cannot afford to fall apart."

Coping mechanisms

This theme reveals the strategies employed by wives of individuals with AUD to manage the challenges posed by their husbands' condition. Extended family support is pivotal, providing emotional and practical assistance that significantly eases the caregiving burden. This support is appreciated and often described as a lifeline, enabling the primary caregiver to manage the dual parenting roles more effectively. Religious and spiritual practices are another critical coping mechanism, offering a profound sense of peace and strength amid the turmoil. These practices are deeply ingrained in the participants' lives, highlighting the role of spirituality in maintaining emotional equilibrium and resilience. Furthermore, establishing routines and boundaries is emphasized to preserve family stability. This approach is instrumental in instilling discipline and structure in the lives of children affected by the unpredictability of a parent's AUD, thereby creating a more predictable and secure environment (Table 3).

**Table 3:** Coping mechanisms

Theme	Sub-theme	Participant Verbatim
Coping mechanisms	Extended family support	"When he is too drunk to notice what is happening around him, my sister-in-law steps in to help with the kids' homework. It feels like having a co-parent when you least expect it, but most need it."
	Religious and spiritual practices	"Every morning, I visit the temple. It is my moment of peace before the storm of the day begins. It is where I gather my strength."
	Establishing routines and boundaries	"Weekends are for family chores and homework, and we stick to that no matter what. It gives the kids a sense of responsibility and normalcy."

Support systems: diverse experiences

This theme sheds light on the critical role of support systems for families impacted by AUD, highlighting the challenges and disparities in accessing such support. Community-based support groups are lauded for their role in creating a sense of community and shared understanding among individuals facing similar challenges, offering crucial emotional support and reducing feelings of isolation. However, the narratives also reveal significant barriers to accessing professional counseling and mental health services, particularly related to affordability and awareness. These barriers are exacerbated by socio-economic and educational disparities, which can prevent the most vulnerable families from obtaining the help they need. This discrepancy underscores the urgent need for more inclusive and accessible mental health services that cater to the diverse needs and financial capabilities of all families dealing with AUD (Table 4).

**Table 4:** Support systems: diverse experiences

Theme	Sub-theme	Participant Verbatim
Support systems: diverse experiences	Community-based support groups	"Hearing stories from other women in the support group, sharing our pains and small victories, makes me feel part of a community that understands what I am going through."
	Access to professional counseling	"I was lucky to find a counselor who offered a sliding scale for payment. It has been transformative for me, but I know many who cannot afford this help."
	Socio-economic and educational disparities	"It is hard when you know there are resources out there that could help, but they are just out of reach because of money or not knowing where to look."

Variation across socio-demographic backgrounds

This theme explores the significant impact of socio-economic status and educational levels on the coping strategies and support systems available to wives of individuals with AUD. The narratives reveal a dichotomy in participants' experiences based on their socio-demographic backgrounds. The reliance on informal support systems is pronounced for those from lower socio-economic backgrounds. Financial constraints and limited access to professional services compel these individuals to turn to their immediate family and community networks for support. This reliance underscores the critical role of these informal networks in providing emotional and practical assistance, highlighting a gap in the accessibility of professional mental health services for the participants. Conversely, participants from higher socio-economic backgrounds demonstrate greater accessibility to and utilization of structured support systems, such as support groups and professional counseling. These individuals often have the financial means and educational awareness to seek out and benefit from professional mental health resources, indicating a disparity in support based on socio-economic and educational differences (Table 5).

**Table 5:** Variation across socio-demographic backgrounds

Theme	Sub-theme	Participant Verbatim
Variation across socio-demographic backgrounds	Reliance on informal support systems	"We lean on each other in the family and the neighborhood. Professional help? That's beyond our reach, financially and otherwise."
	Community and familial networks	"In our community, we talk and share our burdens. It's our way of coping. Going to a counselor is not something we can easily do."
	Accessibility and utilization of structured support systems	"I found a support group for wives of individuals with AUD. It has been enlightening and supportive."
	Awareness and utilization of mental health resources	"Understanding the psychology behind AUD and learning coping strategies through counseling has been invaluable for me."

Role of resilience and adaptation

This theme captures how wives of individuals with AUD manage their challenging circumstances. It underscores resilience as a personal attribute and a dynamic process involving individual and collective efforts to adapt to life's adversities. Participants articulate their resilience journey as a crucial aspect of their adaptation to living with a husband's AUD, emphasizing the necessity of personal growth and the development of inner strength. This journey is not isolated but is deeply interconnected with the welfare of their children, suggesting a reciprocal relationship where the resilience of one family member inspires and strengthens others. Moreover, the narratives reveal a conscious effort by the participants to create a secure and loving environment for their children amid the chaos brought about by AUD. This endeavor goes beyond personal resilience, reflecting a collective approach to adaptation that prioritizes the emotional and psychological well-being of the entire family (Table 6).

**Table 6:** Role of resilience and adaptation

Theme	Sub-theme	Participant Verbatim
Role of resilience and adaptation	Personal growth and resilience journey	"Adapting to this life and finding inner strength are things I have learned over the years. It's hard, but it's necessary."
	Reciprocal nature of resilience	"Seeing my children adapt and stay positive gives me hope. It's what keeps me fighting and adapting in turn."
	Creating a secure and loving environment	"It's not just about me being strong. It's about creating an environment where my children feel secure and loved despite the chaos."

Factors contributing to resilience

This theme encapsulates the diverse and multidimensional sources of strength that enable wives of individuals with AUD to address their challenging circumstances. Social support, both practical and emotional, emerges as a critical factor, with family members and friends playing a pivotal role in providing the necessary assistance and understanding that alleviates the daily challenges faced by these women. Shared experiences with others in similar situations offer a unique form of emotional solace, creating a space for empathy, understanding, and mutual support. This sharing acts as a therapeutic mechanism, reinforcing that they are not alone in their struggles. Community and cultural practices also contribute significantly to resilience, offering a broader support network and a sense of belonging beyond immediate family ties. Engagement in these practices provides spiritual strength and practical assistance, reinforcing the women's sense of identity and support within a larger community. Personal beliefs and values are foundational to resilience, with faith, optimism, and a strong sense of responsibility towards their children driving these women to persevere despite their adversities. This internal motivation is crucial for maintaining hope and strength in the face of ongoing challenges. Moreover, access to educational and financial resources is essential to building resilience. The ability to acquire new skills and achieve financial independence empowers these women to make proactive decisions for their and their children's futures, contributing to a sense of control and self-efficacy (Table 7).

**Table 7:** Factors contributing to resilience

Theme	Sub-theme	Participant Verbatim
Factors contributing to resilience	Social support	"When he loses himself to his drinking, my sister takes the kids to school. It's small, but it's huge for me."
	Shared experiences	"Talking to my friend, who is in a similar situation, makes me feel heard. It's like sharing our load."
	Community and cultural practices	"Being part of the women's group in our temple gives me strength. We support each other beyond just spiritual matters."
	Personal beliefs and values	"Believing that things will get better, that this is a test of my strength, keeps me going."
	Educational and financial resources	"Learning new skills through the community center has given me a way to earn."

## Discussion

The narratives of wives of individuals with AUD in Kerala illuminate the profound impact of AUD on family dynamics, particularly highlighting the augmented parenting roles and responsibilities shouldered by these women. The consistent accounts of increased burdens, the emotional toll on children, and the shift towards solo parenting underscore the multidimensional challenges these wives face [15,16]. As highlighted by the participants, the significant emotional and psychological impact on children further points out the urgency of addressing these issues within the familial context to mitigate long-term adverse outcomes [17,18]. The challenges identified, including financial strain, social stigma, and the resultant stress on the wives, point to AUD's broader societal and economic implications. These challenges affect the immediate family unit and the broader community, highlighting the pervasive nature of AUD's impact [19,20]. The financial hardships and social isolation experienced by these families necessitate a comprehensive approach to support beyond the individual with AUD, encompassing economic support and community-based interventions to reduce stigma and promote inclusion [21].

The participants' coping mechanisms and support systems reveal a spectrum of strategies employed to manage the complexities of their situations. The reliance on extended family, religious practices, and the establishment of routines highlight the adaptive strategies these women use to maintain family stability. However, the variation in coping strategies across socio-demographic backgrounds underscores the influence of socio-economic status and education on the accessibility and utilization of support resources [22]. This variation suggests that interventions must be tailored to meet the diverse needs of families affected by AUD, considering the socio-demographic factors influencing coping and resilience [23,24]. The emphasis on resilience and adaptation in tackling AUD-related challenges within family dynamics is particularly significant. Narratives from the study underscore that resilience is cultivated through a combination of social support, community involvement, personal beliefs, and resource accessibility. This insight is vital for shaping interventions, indicating that enhancing these aspects could bolster resilience in families impacted by AUD. The study suggests various strategies to improve the quality of life for wives of individuals with AUD, such as facilitating access to support groups, community engagement programs, and resources for personal growth [25-27]. These interventions, as highlighted by participants, directly link resilience to the welfare of children, further underlining the importance of supporting the non-AUD husband in their parenting role [28,29]. This approach aims to strengthen family resilience, offering a pathway to better family outcomes irrespective of their socio-economic backgrounds.

Acknowledging the limitations of this study, which include focusing on a specific geographic region and relying on self-reported data, future research should explore these dynamics in diverse contexts and through longitudinal studies to understand the long-term impact of AUD on families. Moreover, there is a need for research that examines the effectiveness of various support interventions, including the potential roles of artificial intelligence (AI), in enhancing resilience and adaptation among families affected by AUD [30]. Understanding the mechanisms through which resilience is developed and sustained, potentially augmented by AI's insights, can inform more targeted and practical support for families navigating the complexities of AUD. This approach ultimately contributes to better outcomes for individuals with AUD and their families.

## Conclusions

This study sheds light on the significant challenges and coping strategies of wives of individuals with AUD, highlighting its profound effects on family dynamics and parenting responsibilities. The findings reveal the heavy burdens, financial hardships, social stigma, and emotional distress faced by these women and their children. Despite such adversities, their resilience and coping mechanisms underscore their determination to uphold family stability and safeguard their children's psychological health. The critical role of extended family, community support, and personal beliefs in addressing the complexities of living with a husband afflicted by AUD is evident. These insights emphasize the urgent need for comprehensive support systems tailored to the multifaceted needs of families impacted by AUD.

Future interventions for the well-being of wives of individuals with AUD should incorporate AI to tailor support effectively. By analyzing socio-demographic factors, AI can offer customized access to professional services, enhance community connections, and provide targeted financial aid, which is crucial to mitigating AUD's impact on families. Moreover, research should aim to create targeted interventions and cultivate a stigma-free environment, thereby supporting the psychological health and overall quality of life of these families. This approach is essential not only for improving individual and familial well-being but also for enhancing community health.
